# Unstructured regions of large enzymatic complexes control the availability of metabolites with signaling functions

**DOI:** 10.1186/s12964-020-00631-9

**Published:** 2020-08-26

**Authors:** Ioannis Skalidis, Christian Tüting, Panagiotis L. Kastritis

**Affiliations:** 1 Interdisciplinary Research Center HALOmem, Charles Tanford Protein Center, Martin Luther University Halle-Wittenberg, Kurt-Mothes-Straße 3a, Halle/Saale, Germany; 2 Institute of Biochemistry and Biotechnology, Martin Luther University Halle-Wittenberg, Kurt-Mothes-Straße 3, Halle/Saale, Germany; 3 ZIK HALOmem, Martin Luther University Halle-Wittenberg, Biozentrum, Room A.2.14, Weinbergweg 22, 06120 Halle/Saale, Germany

**Keywords:** Metabolite, Signaling, Acetyl-coenzyme a, α-Ketoglutarate, Palmitic acid, Disorder, Pyruvate dehydrogenase complex, 2-Oxoglutarate dehydrogenase complex, Fatty acid synthase

## Abstract

**Abstract:**

Metabolites produced via traditional biochemical processes affect intracellular communication, inflammation, and malignancy. Unexpectedly, acetyl-CoA, α-ketoglutarate and palmitic acid, which are chemical species of reactions catalyzed by highly abundant, gigantic enzymatic complexes, dubbed as “metabolons”, have broad “nonmetabolic” signaling functions. Conserved unstructured regions within metabolons determine the yield of these metabolites. Unstructured regions tether functional protein domains, act as spatial constraints to confine constituent enzyme communication, and, in the case of acetyl-CoA production, tend to be regulated by intricate phosphorylation patterns. This review presents the multifaceted roles of these three significant metabolites and describes how their perturbation leads to altered or transformed cellular function. Their dedicated enzymatic systems are then introduced, namely, the pyruvate dehydrogenase (PDH) and oxoglutarate dehydrogenase (OGDH) complexes, and the fatty acid synthase (FAS), with a particular focus on their structural characterization and the localization of unstructured regions. Finally, upstream metabolite regulation, in which spatial occupancy of unstructured regions within dedicated metabolons may affect metabolite availability and subsequently alter cell functions, is discussed.

**Video abstract**

**Graphical abstract:**

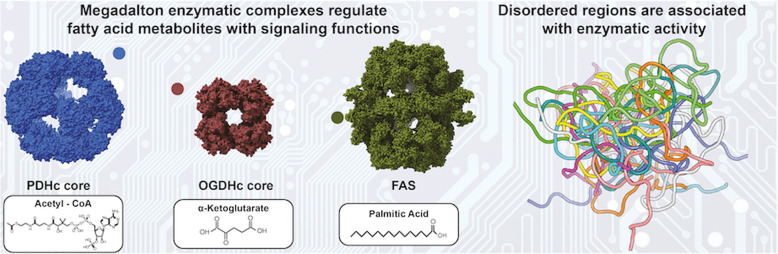

## Background

In the second part of the twentieth century, there have been extensive studies that were mainly focused on discovering the molecular mechanisms behind various diseases, and specifically malignancies. Gene knockout, sequencing and, later, −omics methods, as well as network biology, revealed the different underlying pathways that, when perturbed due to various external or internal factors, are the causes of numerous pathological conditions. This collective work led to the creation of a basic paradigm: a cell will either use an extracellular or intracellular “sensor” to measure changes in the environment and trigger a signaling network. This signal transduction network will propagate via protein-protein interactions, until the signal reaches the nucleus, consequently altering gene expression and triggering a coordinated response to altered conditions, similar to an electric circuit triggered by an on/off switch.

Recent studies, however, have illuminated an aspect of cell signaling that had been mostly overlooked. The effectors of signaling pathways not only can be proteins, but also metabolites, the products or byproducts of cellular metabolism. Earlier observations, e.g., those showing the control of gene expression in bacteria by changes in glucose and lactose concentrations [51] and the covalent modifications of proteins and their implications on protein function in rats [28], hinted towards functions for metabolites in signaling. The current view of cell function is that cellular signaling and metabolism are tightly interconnected [23, 79] and that metabolic networks are as complex as their signaling counterparts; that is, they have an equally intricate role, not only by producing and storing energy or metabolizing proteins, nucleic acids, lipids, and sugars (or to catabolizing them), but also by signaling through their substrates, intermediates, and products to alter cellular behavior and fate.

This review focuses on three different metabolites, acetyl-CoA, α-ketoglutarate and, palmitic acid, and examines how their altered intracellular concentrations can affect various signaling pathways and cellular processes. In addition, more details are presented to offer perspective on the large enzymatic complexes, dubbed “metabolons”, which are the main players in the production and regulation of these metabolites, namely the pyruvate dehydrogenase complex (PDHc), the 2-oxoglutarate dehydrogenase complex (OGDHc), and fatty acid synthase (FAS). Finally, an effort is made to describe unstructured regions in the metabolons and to explain how they may have a potential effect on the availability and regulation of these metabolites.

### Nonmetabolic functions of acetyl-CoA, α-ketoglutarate and palmitic acid

#### Acetyl-CoA is involved in cell fate and survival and the regulation of protein-protein interactions via post-translational modifications

Acetyl-coenzyme A (ACoA) is the lynchpin of cellular metabolism. It is a central component of a plethora of cellular processes, a main intermediate of multiple metabolic pathways and reactions, and the critical cosubstrate of protein acetylation, among other modifications [56, 70]. ACoA is the molecule through which pyruvate is derived from glycolysis before entering the tricarboxylic acid (TCA) cycle and, along with malonyl-CoA, is a precursor for lipid synthesis [5]. ACoA is comprised of an acetyl group that is connected via a thioester bond to coenzyme A, a molecule that in turn is constituted by 3′,5′-ADP, pantothenic acid (a derivative of vitamin B5) and β-mercaptoethylamine. ACoA is produced by multiple sources, e.g., the oxidation of fatty acids and degradation of amino acids, but the most important route of its biosynthesis is through pyruvate generated during glycolysis in a decarboxylation process carried out by PDHc.

**Fig. 1:**
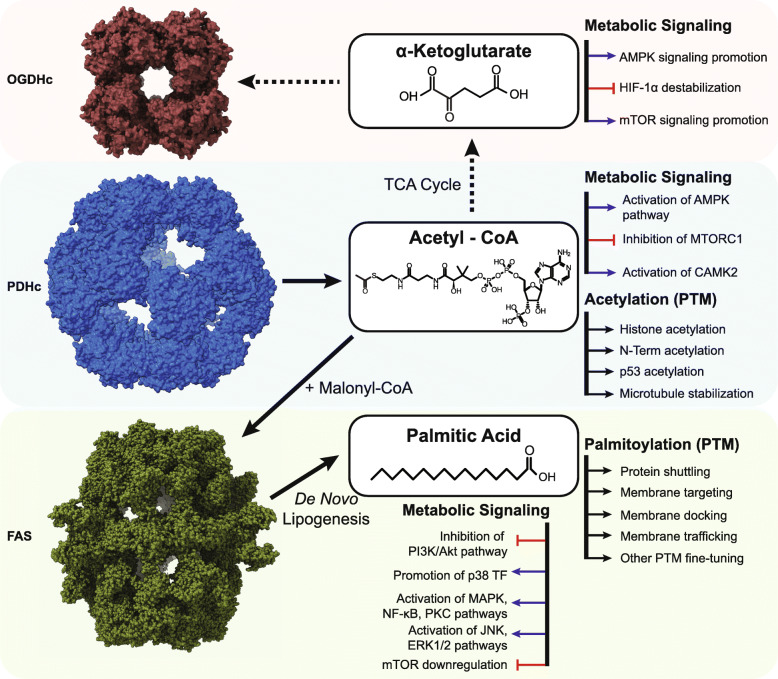
α-ketoglutarate, acetyl-coenzyme A and palmitic acid affect multiple signaling pathways via different mechanisms. On top, α-ketoglutarate is shown which is directly implicated in metabolic pathways and its levels are regulated by 2-oxoglutarate dehydrogenase complex (OGDHc, Electron microscopy data bank ID, EMDB-0108). In the middle, acetyl-coenzyme A is shown, a central metabolite of the cell’s bioenergetic pathways that is produced by the pyruvate dehydrogenase complex (PDHc (EMD-7610)) and affects multiple signaling pathways through different mechanisms. On the bottom, palmitic acid is shown, a main product of de novo lipogenesis, carried out by the fatty acid synthase (FAS, (EMD-4577)) and can influence different signaling targets, directly or through post-translational modifications. Illustrations of the OGDHc E2o core, the PDHc E2p core and the FAS complex were made using ChimeraX (https://www.cgl.ucsf.edu/chimerax/)

Intracellular levels of ACoA are highly regulated, since changes in cytosolic concentration can greatly affect cell fate through multiple processes (Fig. 1). A reduction in cytosolic ACoA has been shown to induce autophagy [67]. The depletion of ACoA activates the AMP-activated protein kinase, which in turn activates Unc-51-like autophagy activating kinase 1 (ULK1), an important kinase with many downstream phosphorylation targets that play critical roles in the formation of the autophagosomes [45]. To further increase the intensity of the signal, reduced cytosolic ACoA levels also result in the inhibition of mechanistic target of rapamycin complex 1 (mTORC1), a known inhibitor of ULK1, thus preventing mTOR signaling, which inhibits autophagy under normal conditions [45]. Additionally, the cytosolic concentration ratio of ACoA and CoA has other important effects on cell death regulation. An increased ratio can induce an ACoA-dependent metabolic signal that activates Ca^2+^/calmodulin-dependent protein kinase II (CaMKII), which in turn phosphorylates caspase-2, through which multiple anti-apoptotic responses are triggered [46].

The most important role of ACoA is arguably as the donor of the acetyl group needed for protein acetylation, which can either occur simultaneously with translation (N-term acetylation) or as posttranslational modifications (PTMs). In the case of N-terminal acetylation, the acetyl group is transferred to the amino group of the first N-terminal residue, usually Ser, Thr, Val, Cys, or Ala, after the initiating Met has been cleaved. This modification contributes not only to protein stability, but also to protein function and localization [60].

Protein acetylation has been extensively studied as a PTM of Lys residues. This addition of an acetyl group to the amino group of a lysine residue has a profound effect on a protein’s stability, catalytic activity, interaction with other proteins and localization (Fig. 1) [64]. Protein acetylation is mediated by two main classes of enzymes, lysine acetyl-transferases (KATs), which add the group to proteins, and lysine deacetylases (KDACs), which reverse the modification by removing the acetyl- group. Histone acetyl-transferases/deacetylases (HATs/HDACs) are critical for modifying histones [63], thus altering the epigenetic profile by either “compacting” or “loosening” different chromatin areas and granting or denying access to the transcriptional machinery of the cell [11]. Other interesting acetylation targets was tubulin subunits that build the microtubule cytoskeleton. Acetylation of Lys40 of α-tubulin is the only PTM that takes part inside the microtubule lumen, in contrast with other PTMs that only affect its outer surface [29]. Even though this kind of modification has been known for some time, its impact is now only being understood, and recent studies have connected it to intracellular trafficking [61], cell migration and autophagy, among other processes [29]. Finally, acetylation is of extreme importance for the function of p53, widely known as “the guardian of the genome” because of its role in tumor suppression. In response to various oncogenic stress factors, p53 activation and the transcription of its downstream targets is directly dependent on its activation by acetylation in multiple Lys sites located at its N-terminal domain [8].

#### α-Ketoglutarate is a promoter of cell survival and regulator of hypoxic signaling pathways

α-Ketoglutaric acid (AKG) (or, as previously known, 2-oxoglutaric acid) is a TCA cycle intermediate that is converted to succinate through decarboxylation by OGDHc. AKG conversion is a rate-determining step of the cycle, as anaplerotic reactions can increase its concentration, using glutamate as a source [25]. AKG is an important substrate for the production of multiple amino acids, such as proline and leucine [50], and in general plays a critical role in cellular metabolic pathways by being an important source of glutamine and glutamate [57].

Apart from its direct role in energy production and biosynthetic pathways, AKG has also been shown to affect cell signaling pathways (Fig. 1) related to tumorigenesis and aging. In the first case, AKG acts as a substrate for prolyl-hydroxylases (PHDs), which constitute a class of α-ketoglutarate-dependent dioxygenases (AKGDDs); these enzymes require AKG and oxygen to hydroxylate hypoxia-inducible factor 1 alpha (HIF-1α) [32], producing succinate in the process. HIF-1α is one of the two subunits (with HIF-1β) of the HIF-1 transcription factor, the main player in cellular oxygen sensing [77]. HIF-1, under hypoxic conditions, activates the transcription of genes related to glycolysis, reducing the dependence on oxidative phosphorylation and enabling the production of ATP in the absence of oxygen. Tumor cells, having to survive in hypoxic conditions, rely heavily on the activation of HIF-1 to satisfy their massive needs for available ATP [68]. In the presence of oxygen, AKG activates prolyl hydroxylase 1–3, which in turn hydroxylates HIF-1α. Through this modification, the von Hippel-Lindau factor recognizes and binds to HIF-1α, marking the protein for proteasome-mediated degradation [49]. Cancer cells take advantage of this process by reducing the levels of available AKG, thus inhibiting the action of AKGDDs, stabilizing HIF-1α and allowing it to migrate to the nucleus, dimerize with HIF-1β and activate the transcription of hypoxia related elements (HREs) [68], thus promoting cell survival and proliferation.

Recent studies have also highlighted the anti-aging effects of AKG [10]. AKG can considerably increase the lifespan and delay the onset of age-related phenotypes of *C. elegans* through downstream inactivation of the TOR signaling pathway. More specifically, ATP synthase, which is a ubiquitous membrane-embedded enzymatic complex, related to energy metabolism and consequently of paramount importance for all cells, was found to be a novel binding target of AKG. AKG acts as an inhibitor of ATP synthase, reducing ATP cell levels, which in turn reduces oxygen consumption and increases autophagy [10]. Additionally, a decrease in ATP levels changes the ADP/ATP ratio in cells, activating 5′ AMP-activated protein kinase (AMPK), which in turn initiates the phosphorylation of the TOR suppressor TSC2, further strengthening autophagy signaling [75].

#### Palmitic acid is a major player in the inflammatory response and regulator of protein localization through palmitoylation

Palmitic acid (PA) is the most common form of saturated fatty acid found inside the human body. ACoA and malonyl-CoA (MCoA) enter the fatty acid synthesis pathway, and MCoA is elongated through a repetitive synthesis cycle until it reaches a length of 16 carbons (16:0). In this process, fatty acid synthase (FAS) is a key enzyme [9]. PA is consistently under tight homeostatic control, and extracellular intake levels are continually balanced by de novo lipogenesis (DNL), hinting at the critical role of PA in various cellular processes [17]. Disturbances to intracellular concentrations can have adverse effects, as it is evident by the implication that PA is involved in various signaling processes associated with cancer phenotypes (Fig. 1). DNL is closely associated with cancer cells through the Warburg effect [18], through which cells show a high preference for anaerobic glycolysis, even before they reach the stage of oxygen deprivation due to reduced access to oxygenated blood.

Excess PA in cells increases diacylglycerol levels through its diversion to nonoxidative metabolic pathways. This diversion, in turn, activates protein kinase C (PKC) and upregulates the phosphorylation of insulin receptor substrate-1 (IRS-1) [52], lowering its activation and subseqently inhibiting the PI3K/AKT signaling pathway, which is a key pathway regulating the cell cycle and is thus directly related to cellular quiescence, proliferation, and cancer. Additionally, PA enhances the p38-mediated activation of PTEN [52], increasing its effect on AKT signaling. In adipose tissue, increased intracellular PA concentrations have been shown to facilitate inflammatory responses, leading to diseases such as insulin resistance and obesity. PA induces the activation of NF-κΒ-, protein kinase C-, and MAP kinase-mediated pathways, which in turn leads to the production of cytokines, such as TNF and interleukin-10, which indicates that cells are in a constant inflammatory state [1]. This inflammatory state is further amplified by the additional phosphorylation of the JNK/ERK kinases, which increase the inflammatory response through their interaction with the MAPK pathway components and can cause pathological conditions such as metabolic syndrome [73]. Interestingly, in the case of hepatocellular carcinoma, high levels of PA have been observed to have antitumor effects by reducing cell proliferation and invasiveness through the downregulation of the mTOR signaling pathway [40].

PA affects cellular processes not only through direct/indirect signaling but by also as the main substrate of another type of posttranslational modification, palmitoylation, through which a palmitate group (a saturated chain of 16 carbons) is attached to a cysteine residue of a protein target via a thioester bond in a process that also has the advantage of being completely reversible [41]. This PTM has been shown to affect numerous functions (Fig. 1), including protein trafficking to the membrane and docking [3]. Specifically, a palmitate group added to a soluble protein can serve as a hydrophobic anchor that facilitates protein docking to a membrane. Additionally, many transmembrane proteins, such as GPCRs, have been shown to undergo palmitoylation, thereby increasing their stability, preventing their aggregation, and modulating their target-specific binding to lipids or other proteins in the membrane [6]. Palmitoylation has also been shown to modulate intercompartmental trafficking in the cell, as in the case of Ras proteins [21], regulating their transfer between Golgi and post-Golgi membrane compartments and further “fine-tune” other PTMs on the same protein, especially those in close proximity to the palmitoylation site [65].

### Metabolons involved in the availability of acetyl-CoA, palmitic acid and α-ketoglutarate

#### Acetyl-CoA: produced by the giant PDHc with > 100 “flexible arms”

The acetylation of CoA is determined by the availability of carbon sources and can have either extramitochondrial or intramitochondrial origins. At low glucose levels, acetyl-CoA synthetase (ACS), with acetate, and alcohol dehydrogenase (ADH), with ethanol, acetylate CoA, coupled with ATP hydrolysis [70]. The degradation of branched-chain ketogenic amino acids (e.g., valine, leucine, and isoleucine) leads to their conversion to α-ketoacids by transamination and eventually to isovaleryl-CoA through oxidative decarboxylation by the mitochondrial branched-chain α-ketoacid dehydrogenase complex (BCKDHc). Isovaleryl-CoA then undergoes dehydrogenation, carboxylation and hydration to form a CoA-derived intermediate before it is cleaved to ACoA and acetoacetate. In mitochondria, also at low glucose levels, the production of ACoA is linked to the β-oxidation of fatty acids. Fatty acids are converted to acyl-CoA and, subsequently, acyl-CoA is degraded in a four-step cycle by four respective enzymes, namely, acyl-CoA dehydrogenase, enoyl-CoA hydratase, 3-hydroxyacyl-CoA dehydrogenase, and thiolase. This enzymatic cycle leads to a new fatty acid chain with two fewer carbons and ACoA as a byproduct. At high glucose levels, glycolysis takes place rapidly, thus increasing the amount of citrate produced by the tricarboxylic acid cycle. Then, citrate is exported from mitochondria to other organelles to be catabolized into ACoA and oxaloacetate by ATP citrate lyase (ACL) coupled with the hydrolysis of ATP. Conversions between pyruvate and ACoA are possible; for example, pyruvate formate lyase converts pyruvate into ACoA and formic acid; however, while ACoA is mainly produced through glycolysis, pyruvate undergoes oxidative decarboxylation, during which it loses its carboxyl group (as carbon dioxide) to become ACoA. This pyruvate dehydrogenase reaction is catalyzed by the pyruvate dehydrogenase complex (PDHc), utilizing pyruvate to produce ACoA, CO_2_, and NADH.

**Fig. 2:**
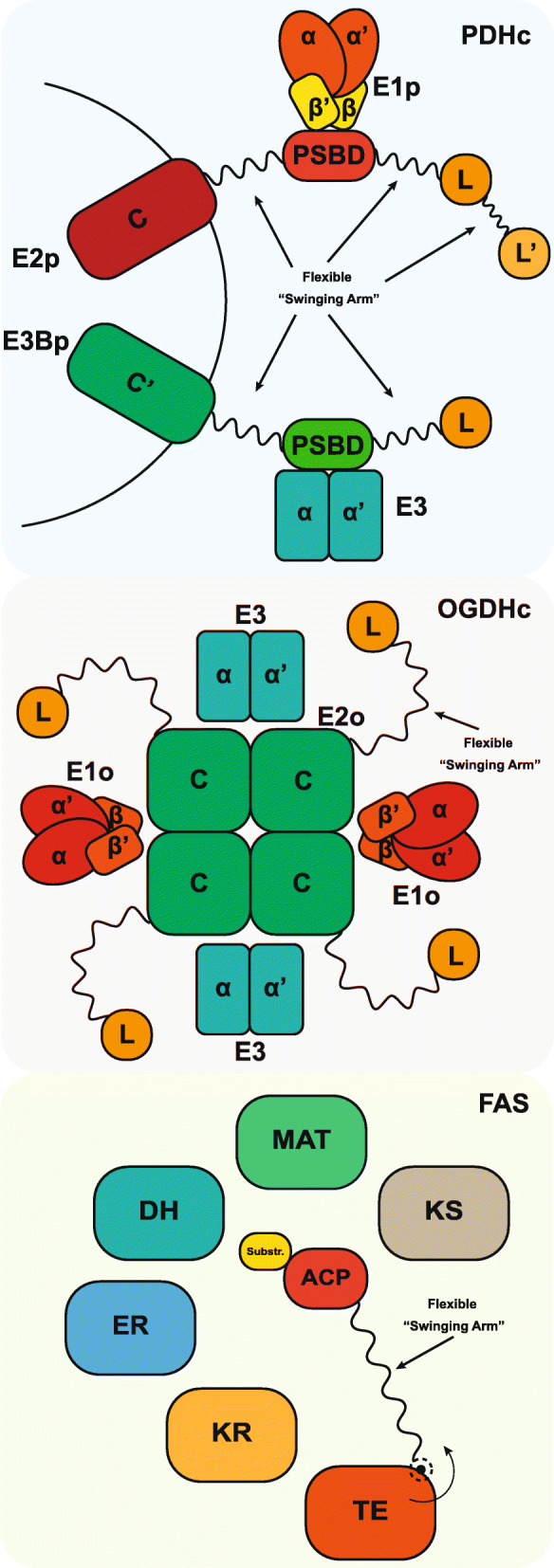
Subunit organization schematic of PDHc, OGDHc and FAS. On top, organization of the different proteins comprising the pyruvate dehydrogenase complex (PDHc) is shown. The flexible linkers connecting the lipoyl domains allow for easier transfer of substrates among the E1p, E2p and E3 subunits needed for the creation of ACoA. E2p: dihydrolipoyl acetyltransferase, E3BP: E3 Binding Protein, C: core domain, PSBD: peripheral subunit binding domain, E1p: pyruvate dehydrogenase (lipoamide), E3: dihydrolipoyl dehydrogenase, L: lipoyl domain. In the middle, organization of the 2-oxoglutarate dehydrogenase complex (OGDHc) is shown. The disordered flexible arms holding the lipoyl domains facilitate the substrate channeling among the core E2o proteins and the peripheral E1o and E3 subunits. E1o: 2-oxoglutarate decarboxylase, E2o: dihydrolipoyl succinyltransferase, E3: dihydrolipoyl dehydrogenase, L: lipoyl domain. On the bottom, subunit organization of the fatty acid synthase (FAS) is shown. The acyl-carrier protein (ACP) is linked to the thioesterase (TE) domain through a flexible disordered arm that allows access to the covalently connected substrate to multiple subunits with different activities needed for de novo lipogenesis. TE: thioesterase, KR: β-ketoacyl reductase, ER: β-enoyl reductase, DH: dehydratase, MAT: malonyl−/acetyl-CoA-ACP-transacylase, KS: β-ketoacyl synthase, ACP: Acyl- Carrier Protein, Substr.: Substrate

**Fig. 3:**
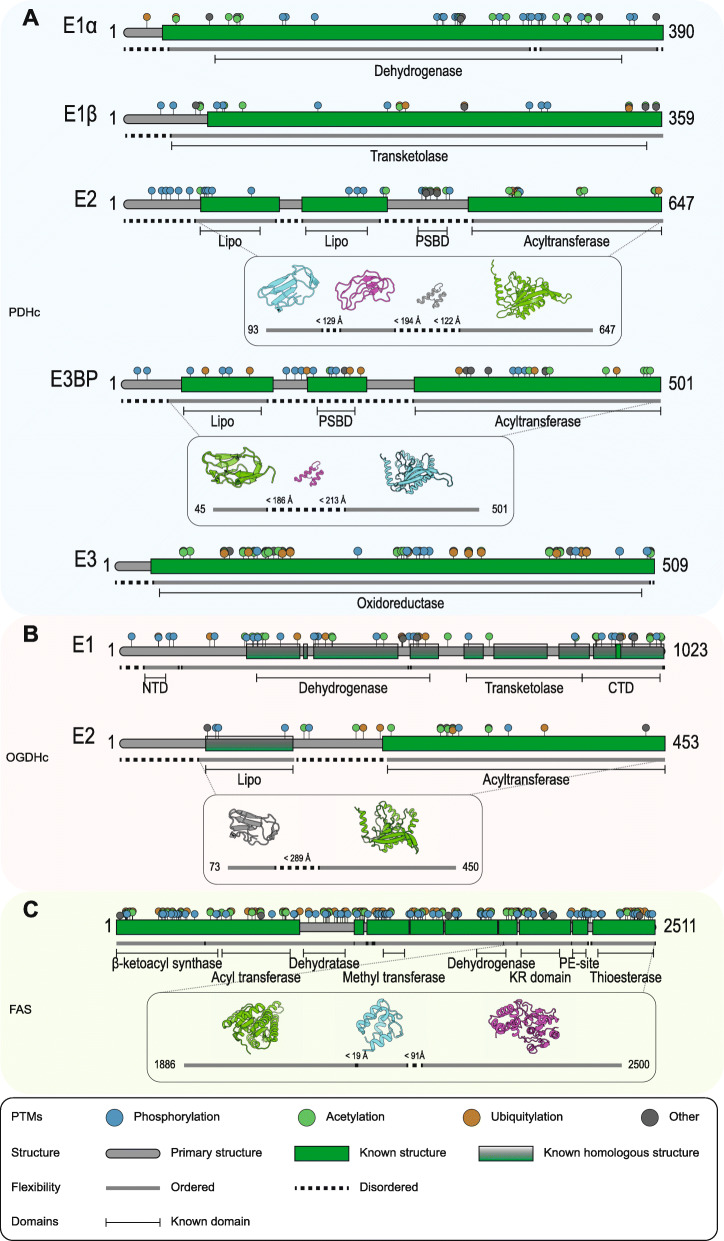
Sequence analysis and characterization of human OGDHc, PDHc and FAS proteins. Protein sequences were analyzed regarding PTMs, known structure, domains and flexibility. Data about PTMs were derived from PhosphoSitePlus (https://www.phosphosite.org/), known structures were datamined from the protein data bank (https://www.rcsb.org/) and manually validated. Homologous structures were identified using HHpred (https://toolkit.tuebingen.mpg.de/tools/hhpred). Domain annotations were derived from the pfam-database (https://pfam.xfam.org/). Disorder prediction was done using SPOT-disorder2 (https://sparks-lab.org/server/spot-disorder2/). Structures shown are in (A) for PDHc E2, the structures of the first (protein data bank accession code, PDB ID: 1FYC) and second (2DNE) lipoyl domain, a placeholder (from E3BP; low homology) PSBD structure (1ZY8) and the catalytic core (6H60) and the lipoyl domain of E3BP (2DNC), the PSBD (1ZY8) and core structure (6H60); in (B) a homologous lipoyl structure from PDHc E2 (1FYC) and the core of OGHDc (6H05); and in (C) the KR-domain (5C37), the acyl-carrier (PE-site; 2CG5) and the thioestarese (4Z49). The maximum distances of the disordered regions are calculated using a Cα-Cα distance of 3.8 Å. Illustrations of 3D protein models were generated using PyMOL (https://pymol.org)

The PDHc is a 10 MDa assembly, one of the largest soluble molecular machineries in the cell. It is composed of three basic enzymes, pyruvate decarboxylase [pyruvate dehydrogenase (lipoamide), E1p], dihydrolipoyl acetyltransferase (E2p) and dihydrolipoyl dehydrogenase (E3) (Fig. 2). In humans, the structural core of the PDHc is formed by multiple copies of the relevant E2p polypeptide chain and is arranged in pseudo-icosahedral (60-mer) symmetry similar to a regular dodecahedron **(**Fig. 1**)**, with each vertex composed of an E2p trimer. The peripheral components E1p and E3 are also present in multiple copies, and bound tightly but noncovalently to the outer region of the core. E2p has a pronounced domain-and-linker structure (Figs. 2 and 3, Additional file 1: Table S1). It is composed of 4 ordered domains connected by flexible linker regions. The 4 ordered domains are directly related to the function of the PDHc, and include, from the N- to the C-terminus, two lipoyl domains (LD1 and LD2; 2× ~ 8 kDa), a structured region critical for binding the peripheral E1p subunits (peripheral subunit-binding domain, PSBD; ∼4 kDa), and the catalytic core module of the E2p (∼26 kDa) (Figs. 2 and 3, Additional file 1: Table S1). Ordered structures of human sequences have been resolved by means of X-ray crystallography [12–14], NMR spectroscopy [33] and cryo-EM [31]. For ACoA production, pyruvate is captured and decarboxylated by E1p and then transferred to a mobile lipoyl domain in E2p. The lipoyl domain transports the acyl group to the E2p catalytic domain, which in turn transfers it to coenzyme A. ACoA is released, and the lipoyl domain of E2p is regenerated (reoxidized) for another cycle by E3. Therefore, the lipoyl domain acts as a “swinging arm” that brings substrates, intermediates and products from one enzymatic center to another. This arm includes the rigid lipoyl domain but is flexible overall, connected by structurally unresolved linker regions [55]. These linker segments in the polypeptide chain are 25–30 amino acids long and act to facilitate the coupling of domain movement with an active site as part of the catalytic mechanism [48]. These linker regions are remarkably predicted to include disorder, at least to a certain extent (Figs. 2 and 3a, Additional file 1: Table S1). Considering fully extended linker regions in the human PDHc (d_Cα-Cα_ = 3.8 Å), the maximum distance between the ordered domains is calculated to be long as 122 Å between the E2p core and the PSBD, 194 Å between PSBD and LD1 and 129 Å between LD1 and LD2. These distances seem very long, being the maximum for extended conformations; Experimental data suggest an overall diameter of the PDHc of ~ 500 Å, with the external diameter in a region of ~ 300 Å, including a gap between the core structure and the areas of external density [39]. Flexible regions are suggested to be extended [39] and highly similar but divergent, with limited immunological cross-reactivity [22]. Due to their high copy number, these regions are likely confined by the spatial proximity of the redundant N-ter domains, to prevent unfavorable van der Waals interactions. The linkers are shown to have disordered structures but are not random coils [59]. The disorder predicted in the flexible parts of the E2p sequence (Fig. 3a, Additional file 1: Table S1) likely contributes to the regulated positioning of the LDs by selective conformational ensembles. For example, mutagenesis or constructs of variable lengths indeed were associated with poor growth rates in bacteria [48]. Considering that 48–60 E2p proteins are present in each PDHc, the extent of the linker regions and the manifested disorder is greatly amplified. The effect of cumulated disordered regions on the resulting conformational ensemble of lipoyl domains is unknown, although the association of molecular redundancy, disordered regions, flexible structural parts, and ordered domains seems to be important. Recently performed molecular dynamics simulations of the full complex suggest significant flexibility changes imposed by neighboring E1p and E3 [27]. Another protein that also contributes to the core structure of the PDHc, the E3 Binding Protein (E3BP), is present in ~ 12 copies, further complicating the ability to predict the overall structure critical for the PDHc reaction. E3BP is very similar to the E2p protein, except that it (a) does not actively acetylate CoA, (b) includes a region that binds specifically the E3 protein but not E1p protein and (c) includes 1 lipoyl domain not 2 lipoyl domains. It is also known to include highly flexible linkers, and those are also predicted to be disordered [7, 24] (Fig. 3a, Additional file 1: Table S1). The overall spatial architecture of the PDHc is highly intricate: 48–60 E1p and ~ 12 E3 proteins surround the E2p-E3BP core (composed of 48–60 E2p and ~ 6–12 E3BP proteins), while > 102 LDs are generally involved. It is reasonable to assume that the disorder manifested in the linker regions promotes the regulation of conformational freedom during the transfer of lipoyl intermediates. Therefore, the generation of ACoA, may depend on the lipoyl domain on and off rates, of its interaction with its surrounding polypeptide chains, and its underlying physical chemical and structural properties.

Another important layer to the regulation of the PDHc is based on posttranslational modifications [53], especially those conferred by its dedicated kinases and phosphatases, which inactivate the PDHc, a phenomenon observed after mitochondrial function is attenuated in cancer [66]. The production of ACoA may be additionally inactivated by phosphorylation of the E1p at distinct Ser sites (Ser-264-α-P, Ser-271-α-P and Ser-203-α-P) present in the two flexible loops (loop A: from 259-α to 282-α, loop B: from 195-α to 205-α), each with a different affinity and specificity [53]. The phosphorylation of loop A plays an integral role in channeling and anchoring thiamine diphosphate to the active site of E1p, whereas the phosphorylation of loop B coordinates a Mg^2+^ ion that is chelated by the diphosphate group of the cofactor [36]. The disorder of these phosphorylation loops was well-defined by the authors, who interpreted the absence of electron density in the crystallographic structures as characteristic of disorder [36, 80]. The phosphorylation of the E1p loops causes a steric clash between introduced phosphoryl groups and nearby residues, abolishing the hydrogen-bonding network essential for maintaining ordered loop conformations [36]. Surprisingly, the increased regulatory loop disorder is associated with the loss of E1p catalytic efficiency, a molecular cause of PDHc deficiency (PDCD) [80]. Other modifications are also important, e.g. Tyr301 phosphorylation [16], which has been implicated as drivers of the Warburg effect and resistance to therapy. Overall, various reproducibly identified PTMs correlate with ordered, but also some with flexible regions, and surprisingly, few regions of predicted disorder in the PDHc are also prone to systematic PTM regulation, with currently unclear physiological effects (Fig. 3a, Additional file 1: Table S1).

Notably, the transamination of the amino acids leucine, isoleucine and valine produces short branched-chain 2-oxo acids that are oxidatively decarboxylated by a single multienzyme complex (BCKDHc) in an intermediate step during ACoA generation. The structure of the BCKDHc is comparable to that of the PDHc and the OGDHc. The OGDHc, a key enzyme in the TCA cycle, converts 2-oxoglutaric acid to acyl-CoA, with the release of CO_2_ and NADH (described below).

#### α-Ketoglutaric acid and the regulation of its availability by the flexible OGDHc

AKG is produced by the oxidative deamination of glutamate by glutamate dehydrogenase and the oxidative decarboxylation of isocitrate by isocitrate dehydrogenase. In addition, alanine transaminase converts AKG and L-alanine to L-glutamate and pyruvate, respectively, in a reversible process. Producing key intermediates in the Krebs cycle, anaplerotic reactions can replenish the cycle after the isocitrate step but before succinyl-CoA step by synthesizing AKG through the transamination of glutamate or through the action of glutamate dehydrogenase on glutamate. Another MDa multienzyme complex, the 2-oxoglutarate dehydrogenase complex (OGDHc), with architecture surprisingly similar to that of the PDHc is involved in the processing of AKG, as described above. It consists of 2-oxoglutarate decarboxylase (E1o), dihydrolipoyl succinyltransferase (E2o) and the same dihydrolipoyl dehydrogenase (E3) enzymes as in the PDHc (Figs. 2 and 3b, Additional file 1: Table S1); these subunits are assembled into a giant 4 MDa complex but with distinct structural alterations, the most prominent of which is the formation of an octahedral E2 core composed of 24 E2 enzymes, not 60 as in the eukaryotic PDHc **(**Fig. 1**)**. In addition, the OGDHc lacks dedicated kinases and phosphatases, and does not have an E3BP protein equivalent to that of the PDHc [69]. The lack of an E3BP and a previously undiscovered mechanism of peripheral subunit recruitment are of particular interest. It is unknown whether a sequence on the outer region of E2o plays a part in the recruitment and spatial confinement of the E1o/E3 proteins. Due to their inherent similarities, one could hypothesize that the mechanism of OGDHc action is similar to that observed for the PDHc and described above. If this mechanism follows the paradigm of the E2p or E3BP proteins, then a disorder-to-order transition likely occurs in the flexible part of the E2o subunit, allowing a similar function and compensating for the absence of a subunit whose role would be homologous to that of the E3BP. If the mechanism is different, then other molecules not yet been discovered may be involved in regulating the proximity of E1o or/and E3 to the E2o, or direct interactions of the peripheral subunits with the E2o might play a role. A recent discovery may shed some light on the topic, as a novel subunit of OGDHc was identified, Kdg4, which has a moonlighting function in mitochondria, being part of both the mitochondrial ribosome and the OGDHc [26]. It binds the E1o-E2o core with the N-ter, and tethers the E3 subunit with its C-terminal domain [26].

Overall, E2o includes a currently structurally resolved E2 acyltransferase domain, which tethers the lipoyl domain to a flexible region, which is predicted to be disordered. Again, the lipoyl arm is tethered to this region, which confers the flexibility to undergo various conformational states and regulate intracomplex interactions [81]. E1o is twice the size of the E1p in the PDHc, combining both dehydrogenase and transketolase functions through an unknown structure. However, E1o undergoes allosteric interactions that control OGDHc activity [54, 58].

The flexible lipoyl arm that putatively attaches to the dehydrogenase domain of the E1o is in a conformational space that enables its interaction with the E1o. Therefore, the key disordered region in the fully extended distance of 289 Å may regulate further processing of the AKG to acyl-CoA, in a similar fashion to that in the PDHc. The same questions regarding the concentrations of multiple flexible regions on the surface of the E2p are also applicable to the OGDHc, where 24 lipoyl domains must be coordinated to perform the transport of reaction intermediates. PTMs have been identified on both the E1o and E2o proteins, and few have been predicted to be localized on corresponding disordered regions (Fig. 3b, Additional file 1: Table S1). It is currently unclear how these PTMs might affect the function and/or dynamics/conformational variation of the flexible sequence. Interestingly, the OGDHc can also act as a posttranslational modifier: by producing succinyl-CoA, the OGDHc can also succinylate fumarase or the PDHc and thus regulate the abundance of other metabolites, such as those in neurons [19].

#### Palmitic acid and FAS form a highly modular enzymatic machine

Palmitic acid (PA) is a component in the body of animals, and in humans, it may constitute as much as 21–30% (molar) of human fat depots. It is also a major lipid component in human breast milk. These roles are based on carbohydrates being converted to PA, the first fatty acid produced during fatty acid synthesis and the precursor to longer fatty acids. As a consequence, PA negatively mediates acetyl-CoA carboxylase (ACC) in a feedback mechanism critical for converting acetyl-CoA to malonyl-CoA, which in turn adds carbons to the growing acyl chain, thus preventing further palmitate generation. The de novo generation of palmitate is controlled by another giant multienzyme machine, the fatty acid synthase complex (FAS, Figs. 1, 2 and 3c), which catalyzes the conversion of acetyl-CoA and malonyl-CoA into long-chain saturated fatty acids in the presence of NADPH [76]. In prokaryotes, plants and mitochondria, fatty acid synthesis consists of 7 structurally independent monofunctional enzymes and an acyl carrier protein. In fungi and animals, however, all of the component enzymes and the acyl carrier protein are fused and organized in large polypeptide chains. FAS is an A_6_B_6_ complex in fungi and an A_2_ complex in humans. In humans, it is a surprisingly long polypeptide chain consisting of ~ 2500 residues with a mass of ~ 273 kDa. The complete structure of human FAS is not yet known, with only fragments of the overall architecture revealed, similar to the previously described PDHc and OGDHc metabolons, despite its overall smaller size (~ 0.5 MDa).

The FAS reaction starts when the acyl moiety of acetyl-CoA is transferred to the acyl carrier protein (ACP) by the malonyl−/acetyl-CoA-ACP-transacylase (MAT). MAT also transacylates the malonyl of the elongation substrate malonyl-CoA to ACP. β-ketoacyl synthase (KS) then causes the decarboxylative condensation of the acyl intermediate with malonyl-ACP converted to β-ketoacyl-ACP (or an acetoacetyl-ACP in the first elongation cycle). The β-carbon is then reduced through β-ketoacyl reductase (KR) in an NADPH-dependent manner, and the product (β-hydroxyacyl-ACP) is dehydrated by a dehydratase (DH) to become a β-enoyl, an intermediate that is then reduced by β-enoyl reductase (ER) in an NADPH-dependent manner to yield a C4 acyl substrate for the subsequent cyclic elongation. Two-carbon units are derived from MCoA until a length of C16 (palmitic acid) to C18 (stearic acid) is reached. The end product is released from the ACP by the thioesterase (TE).

It was previously thought that this cycle was not regulated by FAS itself, and that the binding of ACP to various sites was stochastic and asymmetric, as indicated by molecular dynamics simulation studies [2]. The recent discovery of the γ subunit in yeast FAS showed that FAS enzymatic activity is regulated by the γ subunit in response to an abundance of its cosubstrate NADP and has impacts on the higher-order structure and consequently the conformational space probed by ACP [72]. ACP (residues 2125–2192) is tethered to flexible regions on either side of its termini (residues 2066–2124 at the N-ter, and residues 2125–2241 at the C-ter), which have not yet been captured by crystallography or electron cryomicroscopy at high resolution. Additionally, mobile ACP is not frequently captured in the context of larger FAS structures [35, 38, 72], which points to a significant mobility caused by the connecting flexible regions. These flexible linkers are also predicted to include disordered regions and have only been seen at low resolution with cryo-EM [20]. PTMs are widely present in the FAS structure, but currently their functions are unknown. However, we can still observe various identified PTMs on the ordered, flexible and disordered parts of the polypeptide chain (Fig. 3c).

Considering the similarity in the shuttling of intermediates between FAS, the PDHc and the OGDHc by lipoyl or acyl-carrier arms **(**Fig. 2**)**, tethered to flexible regions with the aim of bringing the substrate from one enzymatic site to the other, it is reasonable to assume that the common feature of the manifested disorder observed in these complexes may contribute to the conformational space that is being explored by the flexible arms. Human and fungal FAS have two and six lipoyl arms, respectively, which are spatially confined, but can intercommunicate within their respective spatial constraints as defined by the length of the tethered flexible linker regions [44] in combination with the suppression of futile catalytic cycles by the γ subunit [72]. The mechanisms of the higher-order regulation of multiple carrier proteins is also currently unknown, since they must be spatially confined in order to avoid unintended protein interactions; in the case of FAS in yeast or *C. thermophilum*, this is succeeded by a chamber-like structure [35, 42], but in the case of its human equivalent, the identity of the regulation mechanisms remains an open question because of the absence of resolved native FAS complexes. In the cases of the PDHc and OGDHc, regulatory confinement may be possible, but a mechanism driving this remains unknown due to the scarcity of structures from native PDHc and OGDHc complexes.

## Conclusions

The evidence shows that multiple metabolites, namely, acetyl-CoA, α-ketoglutarate and palmitic acid, apart from their role in the cellular bioenergetic and biosynthetic pathways, can cause profound changes through their participation in multiple cell signaling pathways [47]. They may affect processes, such as autophagy [4, 67] and inflammation [1, 30], and cell survival and proliferation [70], whereas their perturbation can lead to pathogenic phenotypes, associated with tumor survival and progression [62]. Notably, these specific metabolites can affect the same metabolic pathways, but in different cell types, adding another level of regulation associated with tissue specificity; this is the case of the mTOR signaling pathway [40, 78], although no study has directly investigated this correlation.

All three metabolites investigated in this review are regulated by large and highly intricate enzymatic complexes [34] or “metabolons”, the pyruvate dehydrogenase complex, the 2-oxoglutarate dehydrogenase complex, and fatty acid synthase. Although these complexes may seem rigid and highly ordered, they hide a considerable amount of disorder, mainly in regions where conformational flexibility, one of the characteristics of intrinsically disordered regions, is of paramount importance for the facilitation of substrate accessibility, substrate channeling to multiple enzymes, enhanced protein-protein interactions and increased overall reaction speed [74]. Disordered regions have displayed highly distinct sequence features throughout evolution and, in multiple cases, have been shown to contribute to the aforementioned characteristics, which are needed by protein complexes that require every structural advantage available to optimally regulate the availability of highly important metabolites [15]. The flexible linker regions in PDHc and OGDHc E2 proteins are not conserved regarding sequence and length but retain a very high alanine/proline/serine content. These amino acids often underlie the residue content of the flexible regions. One may speculate that the disordered regions are not based on a highly conserved residue pattern, such as those indicated by active sites, but that the overall composition is critical for their function. Another intriguing feature of the flexible regions of PDHc and OGDHc is a relatively high Lys content (~ 10%). Lys can be spontaneously modified by acetyl-CoA [71], which might thus function as an additional regulator. The overproduction of acetyl-CoA modifies the flexible linker and alters its mobility via a concentration-dependent mechanism, which is not catalyzed by enzymes. Additionally, FAS shows a fascinating evolution path, from a multiprotein complex in bacteria to multidomain organization in mammals, which may correlate with a more controlled mechanism associated with intermediate substrate shuttling and regulation of its activity [2].

Nevertheless, structural studies that delve into disorder of large biomolecular complexes are limited by the very nature of both the disorder and the size of these large enzymatic complexes. Fortunately, advances in the field of structural and computational biology, as demonstrated in the field of electron cryomicroscopy [35], allow us to study protein complexes in their native state [37], preserving the characteristics that are not possible to observe with in vitro protein expression and purification. Studying the organization of the ordered parts of native protein complexes will reveal information on how the flexible, intrinsically disordered regions contribute to regulating enzyme kinetics, protein phosphorylation and disease development. To summarize, understanding the native state of a protein allows us to understand its cell function [43], and when this function is tightly bound to cellular fate, as is the case of ACoA, AKG and PA implicated in multiple cellular processes, insights such as these allow us to elucidate and counteract previously unknown disease mechanisms.

## Supplementary information

**Additional file 1: Table S1.** Domain annotation, location of unresolved, resolved and disordered regions for each subunit of the PDH and OGDH complexes (A) and FAS (B). Each entry is characterized by the number of the starting and ending amino-acid. Methods of annotation of the regions is described in Fig. 3 and its legend.

## Data Availability

Datasets used and/or analyzed during the current study are available from the corresponding author on reasonable request.

## Abbreviations

ACC: Acetyl-CoA carboxylase
ACL: ATP citrate lyase
ACoA: Acetyl-coenzyme A
ACP: Acyl- carrier protein
ACS: Acetyl-CoA synthetase
ADH: Alcohol dehydrogenase
ADP: Adenosine diphosphate
AKG: α-ketoglutaric acid
AKGDD: α-ketoglutarate-dependent dioxygenases
AKT: Protein kinase B
AMPK: 5′ AMP-activated protein kinase
ATP: Adenosine triphosphate
BCKDHc: Branched chain α-ketoacid dehydrogenase complex
CaMKII: Ca^2+^/calmodulin-dependent protein kinase II
CoA: Coenzyme A
cryo-EM: Cryogenic electron microscopy
DH: Dehydratase
DNL: De novo lipogenesis
E1o: 2-oxoglutarate decarboxylase
E1p: Pyruvate dehydrogenase (lipoamide)
E2o: Dihydrolipoyl succinyltransferase
E2p: Dihydrolipoyl acetyltransferase
E3: Dihydrolipoyl dehydrogenase
E3BP: E3 binding protein
ER: β-enoyl reductase
ERK: Extracellular-signal-regulated kinase
FAS: Fatty acid synthase
GPCR: G protein-coupled receptor
HAT: Histone acetyl-transferase
HDAC: Histone deacetylase
HIF-1: Hypoxia-inducible factor
HRE: Hypoxia-response element
IRS-1: Insulin receptor substrate-1
JNK: C-Jun N-terminal kinase
KAT: Lysine acetyl-transferase
KDAC: Lysine deacetylases
KDHc: α-ketoglutarate dehydrogenase complex
KR: β-ketoacyl reductase
KS: β-ketoacyl synthase
LD: Lipoyl domain
MAP: Mitogen-activated protein
MAPK: Mitogen-activated protein kinase
MAT: Malonyl−/acetyl-CoA-ACP-transacylase
MCoA: Malonyl-coenzyme A
mTOR: mechanistic target of rapamycin
mTORC1: mechanistic target of rapamycin complex 1
NADH: Nicotinamide adenine dinucleotide
NADPH: Nicotinamide adenine dinucleotide phosphate
NF-κΒ: Nuclear factor ‘kappa-light-chain-enhancer’ of activated B-cells
NMR: Nuclear magnetic resonance
OGDH: 2-oxoglutarate dehydrogenase
OGDHc: 2-oxoglutarate dehydrogenase complex
PA: Palmitic acid
PDB: Protein data bank
PDH: Pyruvate dehydrogenase
PDHc: Pyruvate dehydrogenase complex
PHD: Prolyl-hydroxylases
PI3K: Phosphatidylinositol 3-kinase
PKC: Protein kinase C
PSBD: Peripheral subunit binding domain
PTEN: Phosphatidylinositol-3,4,5-trisphosphate 3-phosphatase
PTM: Posttranslational modification
RCSB: Research collaboratory for structural bioinformatics
TCA: Tri Carboxylic Acid
TE: Thioesterase
TNF: Tumor necrosis factor
TOR: Target of rapamycin
ULK1: Unc-51 like autophagy activating kinase 1
