# Supplementary material for: Unstructured regions of large enzymatic complexes control the availability of metabolites with signaling functions
Source: Cell Commun Signal. 2020 Aug 26;18:136. doi: 10.1186/s12964-020-00631-9 (PMC7448341; doi:10.1186/s12964-020-00631-9)
Supplement: Supplementary file 2 — Additional file 1: Table S1. Domain annotation, location of unresolved, resolved and disordered regions for each subunit of the PDH and OGDH complexes (A) and FAS (B). Each entry is characterized by the number of the starting and ending amino-acid. Methods of annotation of the regions is described in Fig. 3 and its legend. [file 12964_2020_631_MOESM1_ESM.docx]

| **Α** | **Domain Annotation** | | **Unresolved** | **Resolved** | **Disorder** |
| --- | --- | --- | --- | --- | --- |
|  |  |  |  | | |
| **E1p - alpha** |  |  | 1-28 |  | 1-33 |
|  | 67-360 | Dehydrogenase E1 component |  | 29-390 | 34-293 |
|  |  |  |  |  | 294-302 |
|  |  |  |  |  | 303-385 |
|  |  |  |  |  | 386-390 |
|  |  |  |  | | |
| **E1p - beta** |  |  | 1-56 |  | 1-31 |
|  | 33-349 | Transketolase |  | 57-359 |  |
|  |  |  |  | | |
| **E2p** |  |  | 1-92 |  | 1-87 |
|  | 93-164 | Biotin-requiring enzyme |  | 93-186 | 181-214 |
|  |  |  | 187-213 |  |  |
|  | 220-293 | Biotin-requiring enzyme |  | 214-315 | 306-419 |
|  | 355-389 | E1p binding domain | 316-416 |  |  |
|  | 420-647 | 2-oxoacid dehydrogenases acyltransferase (catalytic domain) |  | 417-647 |  |
|  |  |  |  | | |
| **E3BP** |  |  | 1-56 |  | 1-44 |
|  | 59-130 | Biotin-requiring enzyme |  | 57-141 |  |
|  |  |  | 142-173 |  | 136-272 |
|  | 183-217 | E3 binding domain |  | 174-228 |  |
|  | 274-501 | 2-oxoacid dehydrogenases acyltransferase (catalytic domain) | 229-501 |  |  |
|  |  |  |  | | |
| **E1o** | 48-86 | 2-oxoglutarate dehydrogenase N-terminus | 1-931 |  | 1-47 |
|  | 257-581 | Dehydrogenase E1 component |  |  | 110-118 |
|  | 650-866 | Transketolase, pyrimidine binding domain |  |  | 541-548 |
|  | 869-1014 | 2-oxoglutarate dehydrogenase C-terminal |  | 932-940 |  |
|  |  |  | 941-1023 |  | 1019-1023 |
|  |  |  |  | | |
| **E2o** | 72 - 143 | Biotin-requiring enzyme | 1-217 |  | 1-66 |
|  |  |  |  |  | 147-222 |
|  | 222 - 450 | 2-oxoacid dehydrogenases acyltransferase (catalytic domain) |  | 218-453 |  |
|  |  |  |  | | |
| **E3** |  |  | 1-34 |  | 1-37 |
|  | 43-497 | Pyridine nucleotide-disulphide oxidoreductase |  | 35-509 | 505-509 |

| **Β** |  | **Domain Annotation** | **Unresolved** | **Resolved** | **Disorder** |
| --- | --- | --- | --- | --- | --- |
|  |  |  |  | | |
| **Fatty Acid Synthase** | 1-238 | Beta-ketoacyl synthase, N-terminal domain |  | 1-853 |  |
|  | 243-359 | Beta-ketoacyl synthase, C-terminal domain |  |  |  |
|  | 362-472 | Ketoacyl-synthetase C-terminal extension |  |  | 409-416 |
|  | 493-809 | Acyl transferase domain |  |  |  |
|  | 872-1065 | Polyketide synthase dehydratase | 854-1107 |  | 1105-1109 |
|  |  |  |  | 1108-1151 |  |
|  |  |  | 1152-1166 |  | 1161-1173 |
|  | 1244-1342 | Methyltransferase domain |  | 1167-1362 | 1189-1205 |
|  |  |  | 1363-1367 |  |  |
|  |  |  |  | 1368-1522 |  |
|  |  |  | 1523-1529 |  |  |
|  | 1679-1814 | Zinc-binding dehydrogenase |  | 1530-1774 |  |
|  |  |  | 1775-1779 |  |  |
|  |  |  |  | 1780-1865 | 1801-1804 |
|  |  |  | 1866-1876 |  | 1863-1867 |
|  | 1886-2064 | KR domain |  | 1877-2114 |  |
|  |  |  | 2115-2123 |  | 2117-2121 |
|  | 2126-2187 | Phosphopantetheine attachment site |  | 2124-2194 |  |
|  |  |  | 2195-2217 |  | 2192-2215 |
|  | 2242-2500 | Thioesterase domain |  | 2218-2507 |  |
|  |  |  | 2508-2511 |  | 2506-2511 |

**Table 1. Domain annotation, location of unresolved, resolved and disordered regions for each subunit of the PDH and OGDH complexes (A) and FAS (B).** Each entry is characterized by the number of the starting and ending amino-acid. Methods of annotation of the regions is described in **Figure 3** and its legend.
